# Fecal Carriage of *Staphylococcus aureus* in the Hospital and Community Setting: A Systematic Review

**DOI:** 10.3389/fmicb.2016.00449

**Published:** 2016-05-10

**Authors:** Shantelle Claassen-Weitz, Adebayo O. Shittu, Michelle R. Ngwarai, Lehana Thabane, Mark P. Nicol, Mamadou Kaba

**Affiliations:** ^1^Division of Medical Microbiology, Department of Pathology, Faculty of Health Sciences, University of Cape TownCape Town, South Africa; ^2^Department of Microbiology, Obafemi Awolowo UniversityIle-Ife, Nigeria; ^3^Department of Clinical Epidemiology and Biostatistics, McMaster UniversityHamilton, ON, Canada; ^4^Institute of Infectious Disease and Molecular Medicine, Faculty of Health Sciences, University of Cape TownCape Town, South Africa; ^5^National Health Laboratory Service of South Africa, Groote Schuur HospitalCape Town, South Africa

**Keywords:** carriage, community, fecal, *Staphylococcus aureus*, systematic review

## Abstract

**Background and rationale:** *Staphylococcus aureus* fecal carriage has been identified as a potential source for nosocomial transmission and a risk factor for disease development. This systematic review determined the overall *S. aureus* [including methicillin susceptible and resistant *S. aureus* (MSSA and MRSA)] fecal carriage rates within the community and healthcare settings.

**Methodology:** Peer-reviewed articles indexed in Medline, Scopus, Academic Search Premier, Africa-Wide Information, CINAHL, and Web of Science were identified using applicable and controlled vocabulary through to 11 November 2015. Eligible studies were ascertained by three independent reviewers. Random-effects meta-analyses of proportions were performed to determine *S. aureus*, MSSA and MRSA fecal carriage rates reported by eligible studies.

**Results:** Twenty six studies were included in this review. The pooled estimates for *S. aureus*, MSSA and MRSA fecal carriage were 26% (95% confidence interval (CI): 16.8–36.3%), 86% (95% confidence interval (CI): 65.9–97.9%) and 10% (95% CI: 0.7–27.0%), respectively. Fecal *S. aureus* carriage rates increased on average from 10 to 65% during the first 8 weeks of life, followed by an average carriage rate of 64% at 6 months and 46% at 1 year of life. Genotyping techniques were employed mainly in studies conducted in developed countries and comprised largely of gel-based techniques. Six studies reported on the role of *S. aureus* fecal strains in diarrhea (*n* = 2) and the risk for acquiring infections (*n* = 4). Eight of the 26 studies included in this review performed antibiotic susceptibility testing of *S. aureus* fecal isolates.

**Conclusion:** This study provides evidence that screening for *S. aureus* fecal carriage, at least in populations at high risk, could be an effective measure for the prevention of *S. aureus* transmission and infection in the healthcare and community setting. More well-structured studies need to be conducted and sequence-based genotyping techniques should be employed for the comparison of isolates on a global scale in both developing and developed countries.

## Introduction

*Staphylococcus aureus* is a commensal Gram-positive bacterium, which under certain circumstances may be responsible for pyogenic or toxigenic infections, such as skin and soft tissue infections, toxic shock syndrome and pneumonia (Tong et al., 2015). Its carriage is considered as an important risk factor for subsequent development of hospital and community-acquired infections (Ellis et al., 2004; Wertheim et al., 2004; Maier et al., 2005; Dukic et al., 2013; Levy et al., 2013). The anterior nares is recognized as the primary site for *S. aureus* colonization (Kluytmans et al., 1997; van Belkum et al., 2009; Sollid et al., 2014). Other anatomical niches for *S. aureus* include the skin (Popov et al., 2014), oropharynx (Mertz et al., 2007; Petersen et al., 2013), intestinal tract (Acton et al., 2009), and the vagina (Bourgeois-Nicolaos et al., 2010).

The importance of fecal carriage of *S. aureus* has been recognized more than five decades ago in a study which demonstrated that rectal *S. aureus* carriage preceded those from the nose and throat in new-borns (Hurst, 1960). Thereafter, several studies have provided evidence on the clinical importance of fecal carriage of *S. aureus* [in particular methicillin-resistant *S. aureus* (MRSA)] in the hospital setting (Acton et al., 2009). For example, it has been shown that hospitalized patients with both *S. aureus* fecal and nasal colonization are significantly more likely to have positive skin cultures compared to patients with nasal carriage only (Bhalla et al., 2007). In addition, *S. aureus* fecal carriage may serve as an important source for environmental contamination, which can potentially facilitate nosocomial transmission within the healthcare setting (Bhalla et al., 2007). Furthermore, antibiotic-associated diarrhea attributed to MRSA has also been reported (Lo and Borchardt, 2009; Sizemore et al., 2012; Avery et al., 2015); and patients with MRSA colonized diarrheal stools impact significantly on environmental contamination (Boyce et al., 2007).

Despite the potential role and significance of the sole fecal carriage of *S. aureus* (Lee et al., 1997; Squier et al., 2002; Bhalla et al., 2007) and the transmission dynamics of *S. aureus* in infection, a limited number of studies have focused on fecal *S. aureus* carriage in the hospital and community setting (Acton et al., 2009). This systematic literature review is therefore aimed to determine the overall rate of *S. aureus* [including methicillin susceptible and resistant *S. aureus* (MSSA and MRSA)] fecal carriage amongst individuals in the community and healthcare settings.

## Methodology

This review followed the preferred reporting items for systematic reviews and meta-analyses (PRISMA) guidelines (Moher et al., 2009). The PRISMA check-list for this review is provided in a Supplementary Table (Table S1).

### Literature search strategy

Peer-reviewed articles (written in English and French) published through to 11 November 2015 on *S. aureus* fecal carriage within the community and healthcare settings were evaluated using four electronic databases and a combination of keywords (Table 1). We also explored for additional articles by checking the references cited in the primary eligible studies included in this systematic review.

**Table 1:** **Search strategy performed in four databases**.

**Database**	**Search mode**	**Keywords**
Medline via Pubmed	All fields	(“staphylococcus aureus”) AND (“gut” OR “gastrointestinal” OR “anal” OR “anus” OR “intestinal” OR “rectum” OR “rectal” OR “stool” OR “feces” OR “faeces” OR “fecal” OR “faecal”) AND (“epidemiology” OR “incidence” OR “prevalence” OR “carriage” OR “carriage rate” OR carrier*) AND (“humans” OR “human”)
Scopus via SciVerse	Article title, abstract, keywords	
Academic Search Premier, Africa-Wide Information and CINAHL via EBSCOHost	Boolean/Phrase	
Web of Science via Web of Knowledge	Topic	

### Study selection and data extraction

Potentially relevant articles (selected based on their titles and abstracts) were assessed for eligibility (Table 2) by three independent authors. All potentially eligible articles were screened for “predatory journals” using “Beall's list” (Beall, 2015; Shen and Björk, 2015; Siebert et al., 2015). The corresponding authors of potentially relevant articles were contacted to determine the healthcare exposure status of participants so as to assess their eligibility for inclusion in this systematic review (Table 2). Data extraction was performed independently by two authors using a standardized data extraction form. Disagreements and inconsistencies were resolved by consensus. The following information was extracted from each eligible study: study population, number of participants screened for fecal carriage, participant characteristics (age, health status, exposure to health care settings), sample collection details (sample type, age at which samples were collected, collection site), laboratory techniques (*S. aureus* and MRSA screening methods, genotyping techniques, virulence profile assessment), as well as *S. aureus* and MRSA detection rates.

**Table 2:** **Eligibility criteria**.

**Inclusion criteria for systematic review**	**Exclusion criteria for systematic review**
Studies published from 1920 to 11 November 2015 were included in the search. Studies reporting on *S. aureus* or MRSA carriage from fecal/rectal/anal specimens from humans. Studies providing information on the prevalence of *S. aureus* or MRSA fecal carriage. Healthcare exposure data should include information on whether or not participants were: Hospitalized in the 12 months prior to screening nursing home residents, health care workers, or patients transferred from other hospitals or wards (McKinnell et al., 2013). Screened for *S. aureus* or MRSA fecal carriage within > or ≤48 hours of healthcare contact (Folden et al., 2005; Millar et al., 2007; Otter and French, 2011). Studies published in either English or French.	Studies screening for *S. aureus* or MRSA from samples other than feces/rectal swabs/anal swabs. Fecal samples studied for parasites or bacteria other than *S. aureus*. Articles reporting on the number of *S. aureus* or MRSA isolates detected from fecal specimens or on the number of fecal specimens positive for *S. aureus* or MRSA, but not providing information on the number of participants testing positive for *S. aureus* or MRSA fecal carriage. Studies not providing the necessary healthcare exposure data for participants (via the published article or via correspondence with the authors), in order to categorize participants into Healthy participants, Out-patients, In-patients and Healthcare personnel. Articles published in predatory journals (Beall, 2015). Articles not obtainable from the electronic databases, the University of Cape Town (UCT) library or the UCT inter-library loans.
**Inclusion criteria for meta-analysis of proportions**	**Exclusion criteria for meta-analysis of proportions**
Overall fecal carriage prevalence for *S. aureus* and/or MRSA must be available.	Studies providing fecal carriage rates for participants for which fecal carriage rates have previously been reported. Studies not providing information on the age at which participants were screened. Studies screening a pre-selected group of participants based on microbiological assessments. Studies for which MRSA was not confirmed using molecular methods.

### Operational definitions of terms used in this systematic review

#### Community setting

##### Healthy participants

- Participants reported to be healthy at the time of screening for *S. aureus* or MRSA fecal carriage without any exposure to healthcare settings during the year preceding screening (McKinnell et al., 2013);
- Pregnant women visiting obstetric clinics;
- New-borns and mothers at maternity wards during the time of delivery;
- Mothers and infants reported as healthy at the time of screening for *S. aureus* or MRSA fecal carriage, but exposed to the delivery unit or maternity ward during the year preceding screening.

##### Out-patients

Patients screened for *S. aureus* or MRSA fecal carriage with ≤48 h of healthcare contact (Folden et al., 2005; Millar et al., 2007; Otter and French, 2011). Patients should not have had contact with healthcare settings in the year preceding the study.

#### Healthcare setting

##### In-patients

Patients screened for *S. aureus* or MRSA fecal carriage with >48 h of healthcare contact. Patients screened within ≤48 h after admission should be those transferred from another hospital/ward which will allow for >48 h of hospital contact.

##### Healthcare personnel

Participants screened for *S. aureus* or MRSA fecal carriage working at a healthcare setting with or without any illness.

#### Developed and developing countries

Countries were categorized as developed or developing countries based on data from the International Monetary Fund (http://www.imf.org/external/pubs/ft/weo/2015/01/weodata/groups.htm).

#### Antibiotic susceptibility results

The percentage of isolates (obtained from participants with *S. aureus* or MRSA fecal carriage) resistant to each of the antibiotics assayed was calculated from studies that provided adequate data on antibiotic susceptibility test results. Our review noted susceptibility tests results whether or not the respective studies incorporated published guidelines [such as Clinical Laboratory Standards Institute (CLSI), National Committee on Clinical Laboratory Standards (NCCLS), European Committee on Antimicrobial Susceptibility Testing (EUCAST), Antibiogram Committee of the French Society of Microbiology (CA-SFM), or the Swedish Reference Group for Antibiotics (SRGA) guidelines] in assessing the antibiotic resistance profiles.

### Statistical analysis and data visualization

The *S. aureus*, MRSA and MSSA fecal carriage rates for studies included in this systematic review were calculated as follows:

$$\begin{array}{l} S.\;aureus\text{ fecal carriage rate }(\%)=\\ \;\frac{\text{Participants positive for }S.\;aureus\text{ fecal carriage}}{\text{Participants screened for }S.\;aureus\text{ fecal carriage}}\\ \text{MRSA fecal carriage rate }(\%)=\\ \;\frac{\text{Participants positive for MRSA fecal carriage}}{\text{Participants screened for }S.\;aureus\text{ or MRSA fecal carriage}}\\ \text{MSSA fecal carriage rate }(\%)=\\ \;\frac{\begin{array}{l} (\text{Participants positive for }S.\;aureus\text{ fecal carriage}\\ \text{ - Participants positive for MRSA fecal carriage}) \end{array}}{\text{Participants screened for }S.\;aureus\text{ fecal carriage}} \end{array}$$

Individual reports assessing the same participants for *S. aureus*, MSSA or MRSA fecal carriage were considered as a single report. Calculated fecal *S. aureus* carriage rates were used to derive longitudinal data of individual studies, as well as the average carriage rate amongst these studies, at each time-point. Meta-analyses of proportions were performed to determine the overall *S. aureus*, MSSA and MRSA fecal carriage rates (pooled estimates) among individuals in the community and healthcare settings. Meta-analyses of proportions for MRSA and MSSA did not include studies for which MRSA was not confirmed using molecular methods. For all meta-analyses of proportions, studies screening for MRSA amongst pre-selected vancomycin resistant enterococci (VRE) fecal carriers were excluded. Similarly, meta-analyses of proportions did not include studies that screened for MRSA fecal carriage solely from pre-selected MRSA carriers (MRSA identified from other body sites). Meta-analyses were performed using StatsDirect statistical software version 3.0.165 [England: StatsDirect Ltd. 2016] for studies adhering to the inclusion criteria summarized in Table 2. The StatsDirect statistical software version 3.0.165 [England: StatsDirect Ltd. 2016] was also applied to assess the heterogeneity between the studies included in the meta-analyses (Cochran *Q*-test) (Cochran, 1954) and to determine the inconsistency across the studies included (*I*^2^ statistic) (Higgins et al., 2003). The criterion for statistical significance for the test for heterogeneity was set at alpha = 0.05. The risk of publication bias was assessed and visualized by a Funnel plot (Egger et al., 1997; Sterne et al., 2011).

## Results

### Study selection and characteristics

#### *S. aureus* study selection

**Figure 1:**
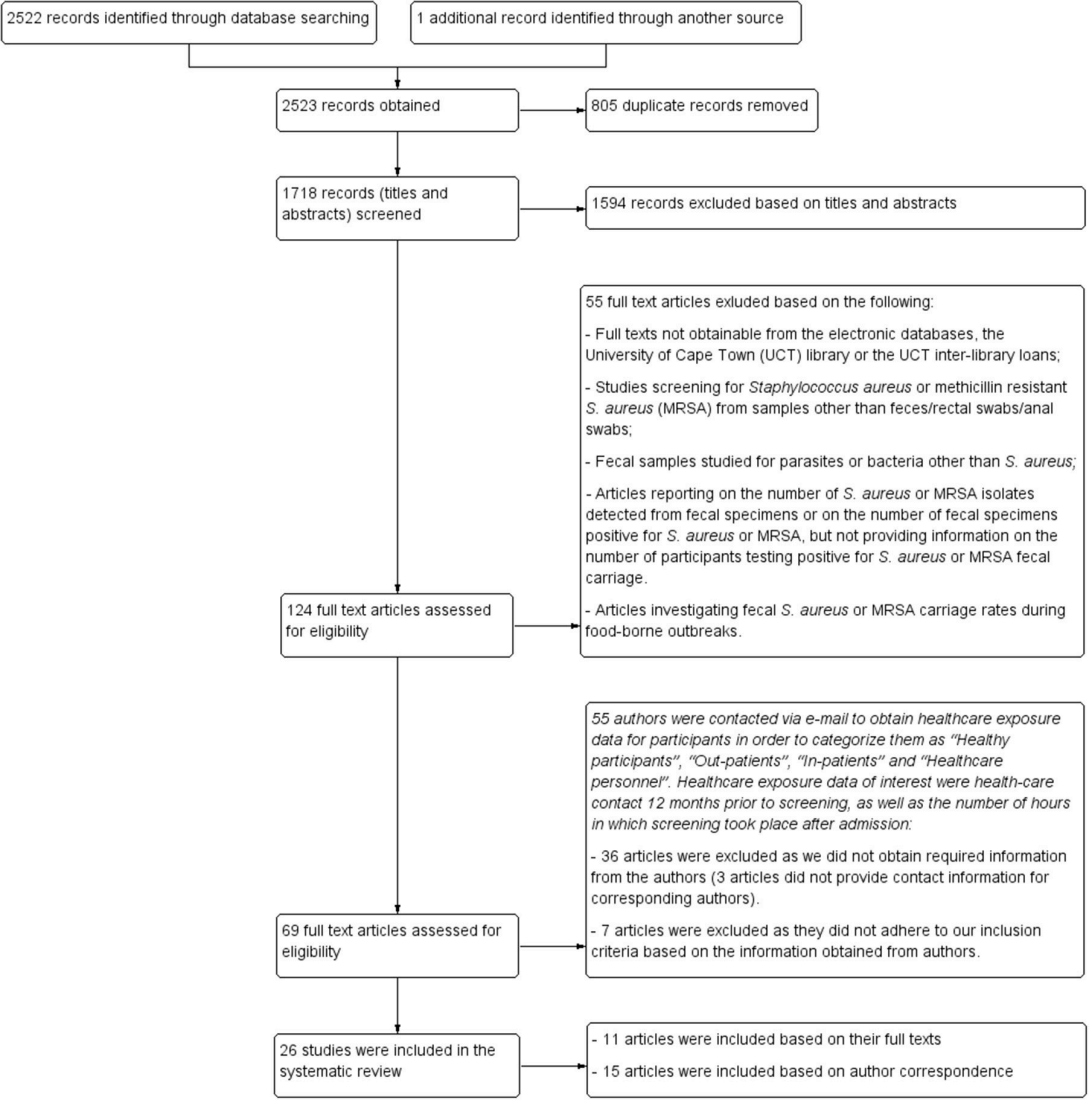
**Study selection**. Flow diagram of identification and selection process for studies included in the systematic review.

Figure 1 outlines the study selection process and the broad reasons for exclusion. The search strategy identified 2522 records. An additional record was identified from the reference list of one of the eligible articles included in the review. A total of 124 potentially eligible reports were identified, of which 69 fulfilled the primary inclusion criteria (Figure 1). The vast majority (80%; 55/69) of these potentially eligible articles did not provide information on healthcare exposure during the year preceding screening and/or did not indicate the duration for which patients were admitted prior to the time of screening. Following correspondence with authors, seven articles were excluded as these reports did not fulfill our inclusion criteria. Moreover, 36 articles were excluded due to lack of required information from corresponding authors or as a result of unavailable author contact information. Consequently, only 26 (11 and 15 reports based on their full texts and information obtained from the authors, respectively) of the 69 studies could be included in our systematic review. The main findings reported by each of the 26 eligible studies are summarized in detail in Tables 3, 4. Select studies that screened for *S. aureus* fecal carriage from both community and healthcare settings are also reported accordingly in Tables 3, 4.

**Table 3:** **Characteristics of eligible studies analysing fecal carriage of *Staphylococcus aureus* or MRSA from the community**.

**Study population setting**	**Participants screened for fecal^Ψ^ carriage (n)**	**Participant characteristics**			**Sample collection**			**Laboratory technique(s)**				***S. aureus* detection**	**MRSA detection**	**References**
		**Age range**	**Health status**	**Exposure to healthcare setting 12 months prior to screening^†^**	**Sample type**	**Age at which samples were collected**	**Site at which samples were collected**	***S. aureus* detection**	**MRSA detection**	**Genotyping**	**Virulence profile analysis**	**% (n/N)**	**& (n/N)**	
**CATEGORY: HEALTHY PARTICIPANTS**														
Italy	100	Birth to 12 months	Healthy	At time of delivery	Rectal swabs	3 days	Delivery unit	Phenotypic	NP	RAPD	SE TSST	5 (5/100)^φ^	NA	Lindberg et al., 2010
					Feces	1 week	Home					15 (15/100)^φ^		
						2 weeks						24 (24/100)^φ^		
						4 weeks						34 (34/100)^φ^		
						8 weeks						45 (45/100)^φ^		
						6 months						52 (52/100)^φ^		
						1 year						31 (27/86)^φ^		
						Overall prevalence						66 (66/100)^φ^		
Mozambique	121	≤14 days to 1 year	Apparently healthy	NR	Feces	≤14 days to 1 year	Home	Molecular	NP	NP	NP	77 (92/120)	NA	González et al., 2013
Nigeria	120	15–35 years	Healthy	No	Feces	15 to 35 years	Provided by participants	Phenotypic	Phenotypic	NP	NP	32 (38/120)	34 (13/38)^¶^	Onanuga and Temedie, 2011
Spain	21	7–35 days	Healthy	At time of delivery^φ^	Feces	1 week 2 weeks	Home	Phenotypic and Molecular	Phenotypic and Molecular	*spa* typing *agr* typing	SE PVL	10 (2/21) 14 (3/21)		Benito et al., 2015
						5 weeks				MLST	ET	48 (10/21)		
						Overall prevalence				PFGE	TSST	57 (12/21)	42 (5/12)	
											AUR			
											BAP			
											CNA			
Spain	100	2–89 years	Healthy	No^φ^	Feces	2–89 years	NR	Phenotypic and Molecular	Phenotypic and Molecular	*spa* typing *agr* typing	SE PVL	15 (15/100)	0 (0/15)	Benito et al., 2013
										MLST	ET			
											TSST			
											AUR			
											BAP			
											CNA			
Spain	50	7–23 months	Healthy	At time of delivery^φ^	Feces	7–23 months	Nurseries	Phenotypic and Molecular	Phenotypic and Molecular	NP	NP	6 (3/50)	0 (0/3)	Domínguez et al., 2002
Sweden	100	Birth to 12 months	Healthy	At time of delivery	Rectal swabs	3 days	Delivery unit	Phenotypic	NP	RAPD	SE TSST	16 (16/100)^φ^	NA	Lindberg et al., 2010
					Feces	1 week	Home					48 (48/100)^φ^		
						2 weeks						56 (56/100)^φ^		
						4 weeks						64 (64/100)^φ^		
						8 weeks						72 (72/100)^φ^		
						6 months						68 (68/100)^φ^		
						1 year						55 (55/100)^φ^		
						Overall prevalence						78 (78/100)^φ^		
Sweden	64	Birth to 8 weeks	Healthy	At time of delivery	Rectal swabs	3 days^φ^	Delivery unit^φ^	Phenotypic	NP	NP	SE TSST	13 (8/64)^φ^	NA	Lundell et al., 2009
					Feces	1 week	Home^φ^					39 (25/64)^φ^		
						2 weeks						53 (33/62)^φ^		
						4 weeks						59 (37/63)^φ^		
						8 weeks						71 (44/62)^φ^		
						Overall prevalence						73 (47/64)^φ^		
Sweden	50	Birth to 12 months	Healthy	At time of delivery	Rectal swabs	3 days	Delivery unit	Phenotypic	NP	RAPD	ET	20 (10/50)	NA	Lindberg et al., 2004a
						1 week	Home			PFGE	TSST	40 (20/50)		
						2 weeks						52 (26/50)		
						4 weeks						60 (30/50)		
						8 weeks						64 (32/50)		
						Overall prevalence						68 (34/50)		
	37	Apparently healthy^φ^	Allergic and non-allergic mothers	At time of delivery	Feces	1 week after delivery or at a later stage	Home					24 (9/37)	NA	
Sweden	81	Birth to 12 months	Healthy	At time of delivery	Rectal swabs	3 days	Delivery unit at 3 days and home at 1 week to 1 year	Phenotypic	Phenotypic	RAPD	ET TSST	NR	0 (0/81)^φ^	Lindberg et al., 2004b
						1 week								
						2 weeks								
						4 weeks								
						8 weeks								
						6 months								
						1 year								
Sweden	49	Birth to 12 months	Healthy	At time of delivery	Rectal swabs	3 days	Delivery unit	Phenotypic	NP	RAPD	ET TSST	16 (8/49)	NA	Lindberg et al., 2000
					Feces	1 week	Home					57 (28/49)		
						2 weeks						65 (32/49)		
						4 weeks						65 (32/49)		
						8 weeks						73 (36/49)		
						6 months						73 (36/49)		
						1 year						53 (26/49)		
						Overall prevalence						86 (42/49)^φ^		
United Kingdom	30	2–7 months	Healthy	NR	Feces	2 weeks	Home	Phenotypic	NP	NP	SE TSST	37 (11/30)	NA	Harrison et al., 2009
						10 weeks						40 (12/30)		
						7 months						40 (12/30)		
						Overall prevalence						40 (12/30)		
United States of America	147	>18 years	Healthy pregnant women at 35-37 weeks of pregnancy	No^φ^	Rectal swabs	>18 years	Obstetric clinics	Phenotypic	Phenotypic and Molecular	SCC*mec* typing PFGE	PVL	4 (6/147)^φ^	0 (0/6)^φ^	Andrews et al., 2009
United States of America	38	1 day to 2 weeks	Healthy	At time of delivery	Feces	1–2 days	New-born unit	Phenotypic	Phenotypic and Molecular	PFGE	PVL	0 (0/38)	0 (0/38)	Gries et al., 2009
						2 weeks						26 (6/23)	33 (2/6)	
						Overall prevalence						26 (6/23)	33 (2/6)	
**CATEGORY: OUTPATIENTS**														
India	100	16–88 years	Patients at admission	No long hospital stay or admission to other hospitals	Feces	16–88 years	Hospital	Phenotypic	NP	NP	NP	0 (0/100)	NA	Deepa et al., 2014
Jordan	216	≤28 days to 1 year	NR	No	Feces	≤28 days to 1 year	Clinic	Phenotypic	Phenotypic	SCC*mec* typing	ET	17 (37/216)	59 (22/37)	Shehabi et al., 2013
											PVL			
											SE			
											TSST			
Nigeria	1761	≤5 years	Diarrhoeic children	No^φ^	Feces	< 1 year	Hospital	Phenotypic	Phenotypic	NP	ET	3 (11/416)	NR	Efuntoye and Adetosoye, 2003
						1.1–2.0 years						4 (13/323)	NR	
						2.1–3.0 years						4 (12/309)	NR	
						3.1–4.0 years						5 (15/292)	NR	
						4.1–5.0 years						5 (21/421)	NR	
						Overall prevalence						4 (72/1761)	NR	
Saudi Arabia	58	NR	Patients at admission (< 48 h)^φ^ with diarrhea or abdominal pain	No^φ^	Feces	NR	Hospital	Phenotypic	Phenotypic	NP	NP	NA	9 (5/58)	Babay and Somily, 2009
United States of America	150	Birth to 18 years	Children requiring abscess drainage (n = 60)	No^φ^	Rectal swabs	Birth to 18 years	Hospital	Phenotypic and Molecular	Molecular	MLVA SCC*mec* typing	PVL	47 (28/60)	NR	Faden et al., 2010
			Children requiring general surgery (n = 90)									1 (1/90)	NR	

Ψ Fecal samples, rectal swabs, anal swabs, peri-rectal or peri-anal swabs.

† Hospital, long-term care facility, nursing homes, maternity wards.

¶ Resistant to cefoxitin.

φ Information obtained from the author.

Phenotypic identification: culture characteristics on mannitol salt agar, Baird-Parker agar, Tripticase soy agar, Chapman agar, Staphylococcus medium 110, positive results for Gram stain, catalase, coagulase and DNase Tests.

agr, Accessory gene regulator; AUR, Aureolysisn; BAP, biofilm-associated protein; CAN, collagen-binding protein; ET, Exfoliative toxins; MLVA, Multiple-locus variable-number tandem repeat analysis; MRSA, methicillin resistant Staphylococcus aureus; NR, Not reported; NP, Not performed; NA, Not applicable; PFGE, pulsed-field gel electrophoresis; PVL, Panton-Valentine Leukocidin; RAPD, random amplified polymorphic DNA; SCCmec, staphylococcal cassette chromosome mec; SE, Staphylococcal enterotoxins; spa, Staphylococcus aureus protein A; TSST, Toxic shock syndrome toxin.

**Table 4:** **Characteristics of eligible studies analysing fecal carriage of *Staphylococcus aureus* or MRSA from the healthcare setting**.

**Study population setting**	**Participants screened for fecal^Ψ^ carriage (n)**	**Participant characteristics**			**Sample collection**			**Laboratory technique(s)**				***S. aureus* detection**	**MRSA detection**	**Reference**
		**Age range**	**Health status**	**Exposure to healthcare setting 12 months prior to screening^†^**	**Sample type**	**Age at which samples were collected**	**Site at which samples were collected**	***S. aureus* detection**	**MRSA detection**	**Genotyping**	**Virulence profile analysis**	**% (n/N)**	**& (n/N)**	
**CATEGORY: IN-PATIENTS**														
France	748	Mean age: 55 years±12	Liver cirrhosis	Hospitalized for minimum of 2 weeks	Feces	Mean age: 55 years±12	Hospital	Phenotypic	Phenotypic	NP	NP	NR	12 (93/748)	Campillo et al., 2001
France	327	NR	Chronic liver disease, post-surgical patients, patients with alcohol withdrawal and digestive tract diseases	Patients transferred from other hospitals	Feces	NR	Hospital	Phenotypic and Molecular	Phenotypic	PFGE	NP	NR	11 (36/327)	Dupeyron et al., 2002
Germany	2727	NR	Nosocomial diarrhea	≥72 h at the time of study	Feces	NR	Hospital	Phenotypic	Phenotypic	NP	SE	7 (198/2727)	15 (29/198)	Flemming and Ackermann, 2007
Germany	131	NR	NR	Inpatients positive for MRSA	Rectal swabs	NR	Hospital	Phenotypic and Molecular	Phenotypic and Molecular	PFGE	SE	NR	47 (61/131)	Klotz et al., 2005
Jordan	214	≤28 days to 1 year	NR	NR	Feces	≤28 days to 1 year	NICU	Phenotypic	Phenotypic	SCC*mec* typing	ET	2 (5/214)	20 (1/5)	Shehabi et al., 2013
											PVL			
											SE			
											TSST			
Saudi Arabia	122	NR	NR	≥72 h at the time of study	Feces	NR	Hospital	Phenotypic	Phenotypic	NP	NP	NA	7 (9/122)	Babay and Somily, 2009
United States of America	810 (2000-01)	NR	Cancer	Inpatients^φ^	Rectal swabs	NR	Hospital	Phenotypic and Molecular	Phenotypic	*spa* typing	PVL	NR	0.6 (5/810)^φ^	Srinivasan et al., 2010
										MLST				
	925 (2006-07)									PFGE			2.9 (27/925)^φ^	
United States of America	161	57–103 years	Fecal and urinary in-continence, pressure ulcers, diabetes, COPD, heart failure	Long-term care facility residents	Rectal swabs	57-103 years	Long-term care wards	NP	Phenotypic	PFGE	NP	NA	3 (4/161)	O'Fallon et al., 2009
United States of America	37	48–91 years	Chronic renal failure, diabetes, chronic dermato-logic infections	Inpatients positive for VRE	Feces	48 to 91 years	Hospital	Phenotypic	Phenotypic	PFGE	NP	62 (23/37)	87 (20/23)	Ray et al., 2003
United States of America	114	NR	NR	Skilled-care patients admitted for long-term care	Rectal swabs		Hospital	Phenotypic	Phenotypic	PFGE	NP	NR	5 (6/114)	Trick et al., 2001
**CATEGORY: HEALTHCARE PERSONNEL**														
United States of America	62	>18 years	Healthy pregnant women at 35–37 weeks of pregnancy	No^φ^	Rectal swabs	> 18 years	Obstetric clinics	Phenotypic	Phenotypic and Molecular	SCC*mec* typing	PVL	3 (2/62)^φ^	0 (0/2)^φ^	Andrews et al., 2009
										PFGE				
United States of America	55	36 years±11	NR	Nurses (n = 29), Physicians (n = 15), Others (n = 9), Unknown (n = 2) (mean patient contact years: 13 years±9)	Fecal	36 years±11	NR	NP	Phenotypic	NP	NP	NA	0 (0/55)	Carmeli et al., 1998

Ψ Fecal samples, rectal swabs, anal swabs, peri-rectal or peri-anal swabs.

† Hospital, long-term care facility, nursing homes, maternity wards.

φ Information obtained from the author.

Phenotypic identification: culture characteristics on mannitol salt agar, Baird-Parker agar, Tripticase soy agar, Chapman agar, Staphylococcus medium 110, positive results for Gram stain, catalase, coagulase and DNase Tests.

agr, Accessory gene regulator; AUR, Aureolysisn; BAP, biofilm-associated protein; CNA, collagen-binding protein; COPD, chronic obstructive pulmonary disorder; ET, Exfoliative toxins; MLVA, Multiple-locus variable-number tandem repeat analysis; MRSA, methicillin resistant Staphylococcus aureus; NICU, Neonatal intensive care unit; NR, Not reported; NP, Not performed; NA, Not applicable; PFGE: pulsed-field gel electrophoresis; PVL, Panton-Valentine Leukocidin; SCCmec, staphylococcal cassette chromosome mec; SE, Staphylococcal enterotoxins; spa, Staphylococcus aureus protein A; TSST, Toxic shock syndrome toxin; VRE, Vancomycin-resistance enterococci.

#### Characteristics of reports from community and healthcare settings

##### Reports on *S. aureus* fecal carriage

A total of 19 reports investigated fecal *S. aureus* carriage within the community setting, of which five and 14 studies reported on fecal carriage rates from outpatients and healthy participants, respectively (Table 3). Moreover, the majority (64%; 9/14) of reports on fecal *S. aureus* carriage rates from healthy participants were of longitudinal design and investigated infants up until one year of age (Table 3). Of the five reports on fecal carriage rates from outpatients, a single study performed a longitudinal analysis of *S. aureus* fecal carriage (Efuntoye and Adetosoye, 2003) and another investigated infants during the first year of life (Shehabi et al., 2013). Study sizes for the community setting ranged between 21 and 1761 participants (Table 3).

Fecal *S. aureus* carriage within the healthcare setting was noted in 12 reports (Table 4). Of these, 10 were from inpatients and two from healthcare personnel. All reports on inpatients were of cross-sectional design and the majority (60%; 6/10) did not provide information on the age of the participants. In addition, the two studies on healthcare personnel were cross-sectional in design and carried out in the United States of America (USA) (Carmeli et al., 1998; Andrews et al., 2009). Study sizes for healthcare-based reports ranged between 37 and 2727 participants (Table 4).

##### Reports on methicillin susceptible and resistant *S. aureus* fecal carriage

Six of the 19 reports on *S. aureus* fecal carriage from the community setting provided MRSA fecal carriage rates confirmed by molecular methods (Table 3). Five of these studies (conducted in developed countries) reported both *S. aureus* and MRSA fecal carriage rates which allowed for the calculation of MSSA fecal carriage rates. Only one study within the healthcare setting (conducted in the USA) confirmed fecal MRSA carriage by screening specimens using a molecular approach (Andrews et al., 2009).

### Pooled estimates of *S. aureus* fecal carriage rates assessed by meta-analyses

**Figure 2:**
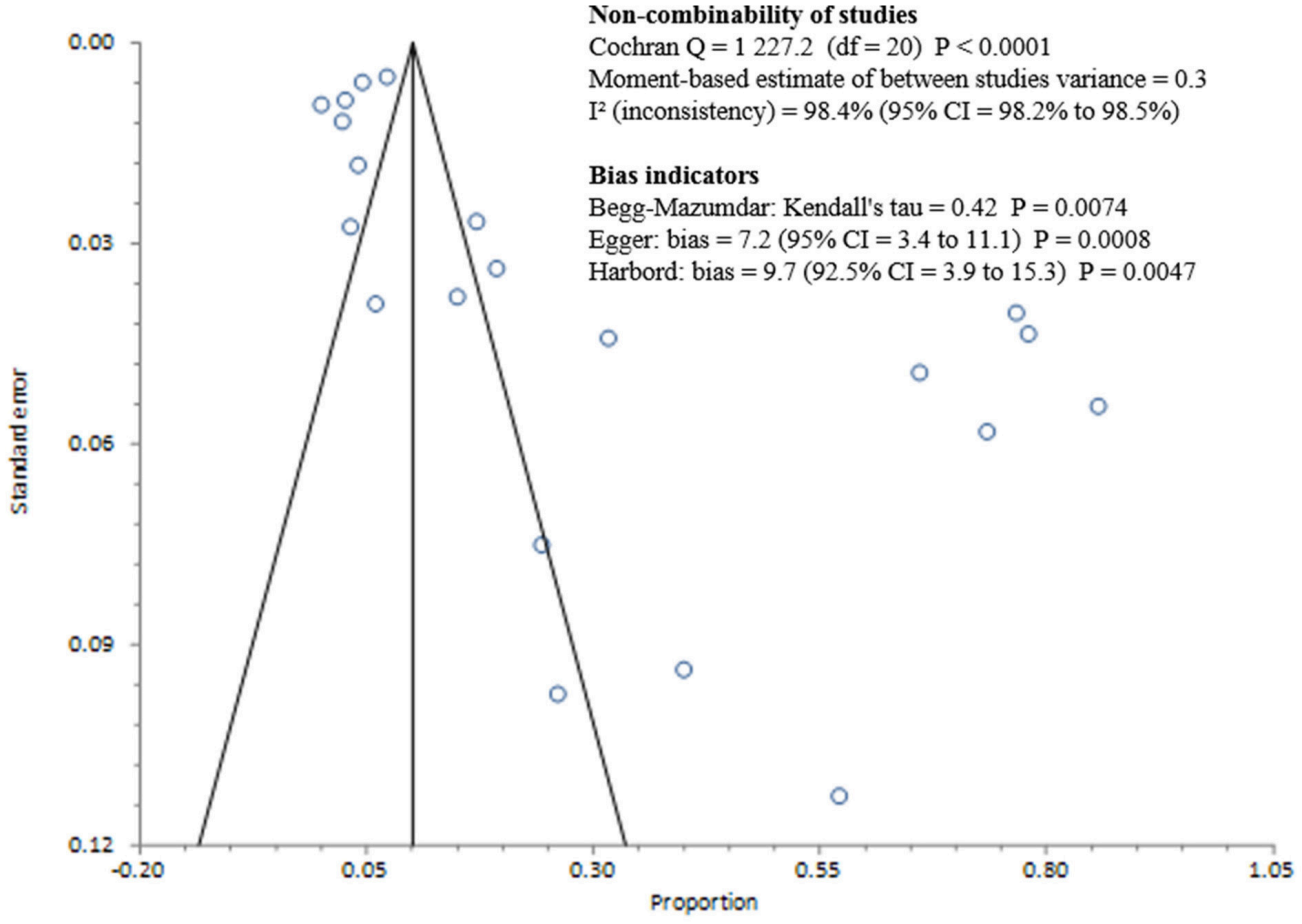
**Bias assessment (Funnel) plot for studies assessing *Staphylococcus aureus* fecal carriage rates**.

**Figure 3:**
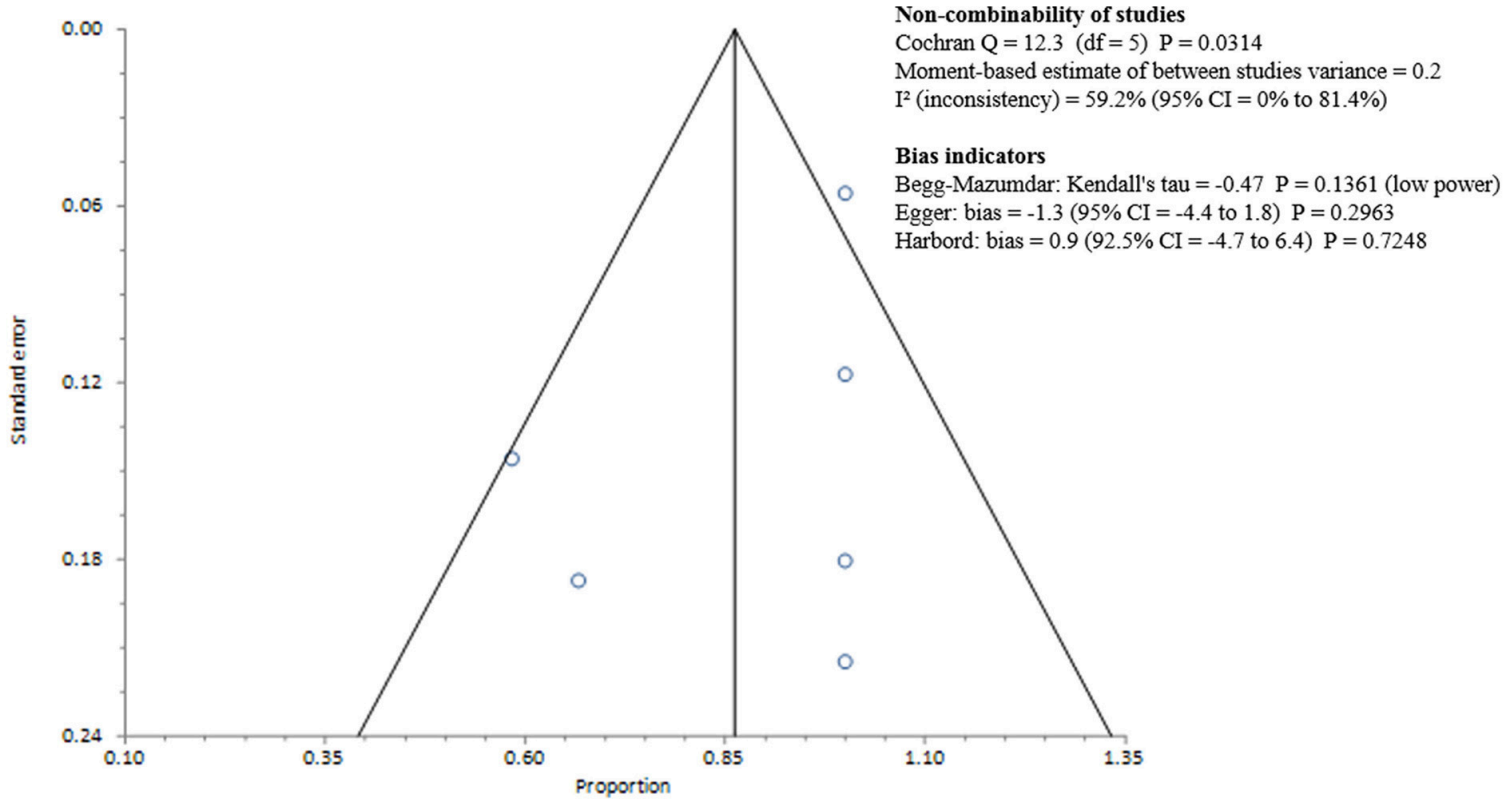
**Bias assessment (Funnel) plot for studies assessing Methicillin susceptible *Staphylococcus aureus* fecal carriage rates**.

**Figure 4:**
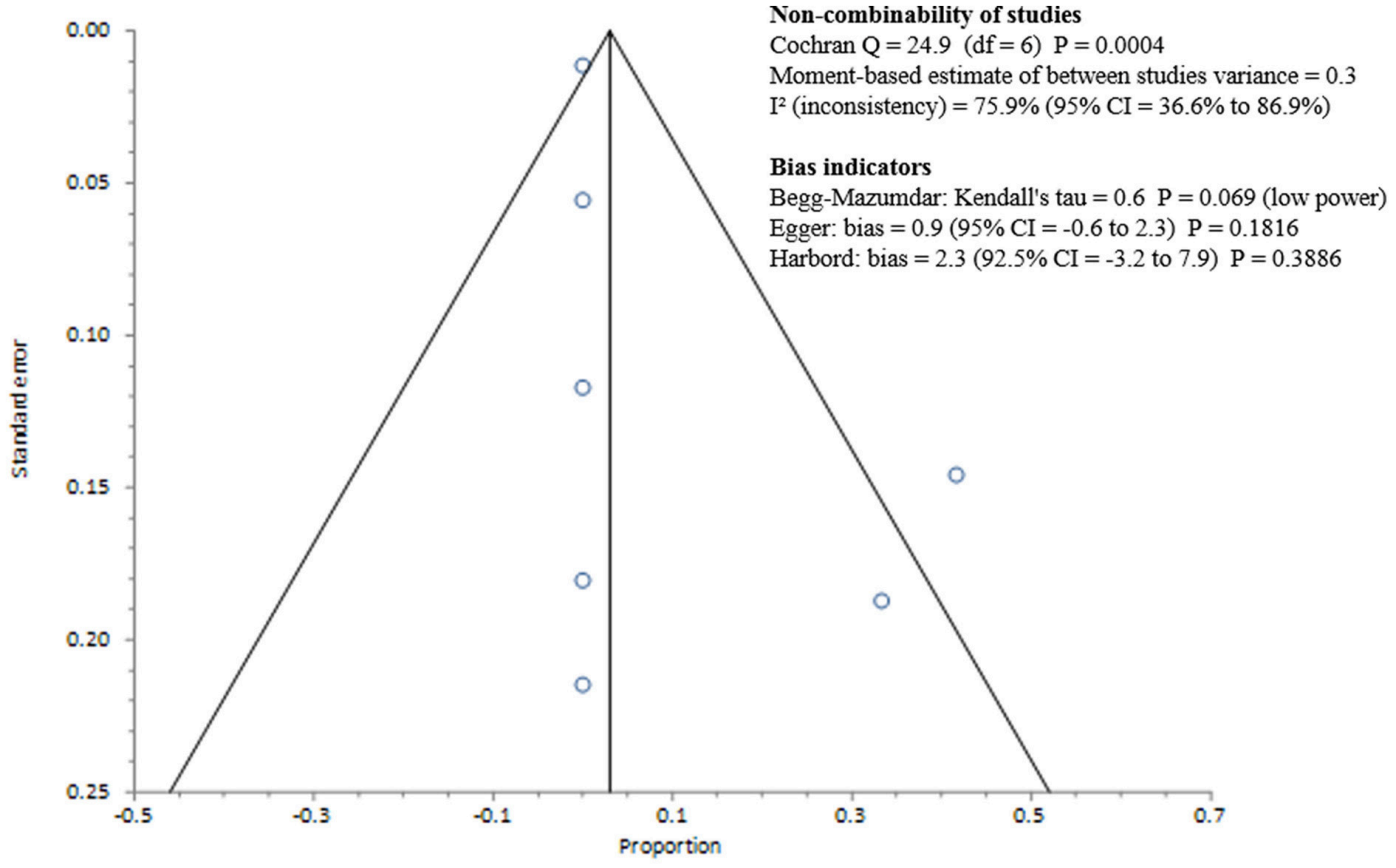
**Bias assessment (Funnel) plot for studies assessing Methicillin resistant *Staphylococcus aureus* fecal carriage rates**.

Studies included in all of the proportional meta-analyses were heterogeneous, as determined by the Cochrane Q test and *I*^2^ statistic (Figures 2–4). We could not determine pooled MSSA or MRSA fecal carriage rates within the healthcare setting as only a single study was considered eligible for this analysis.

**Figure 5:**
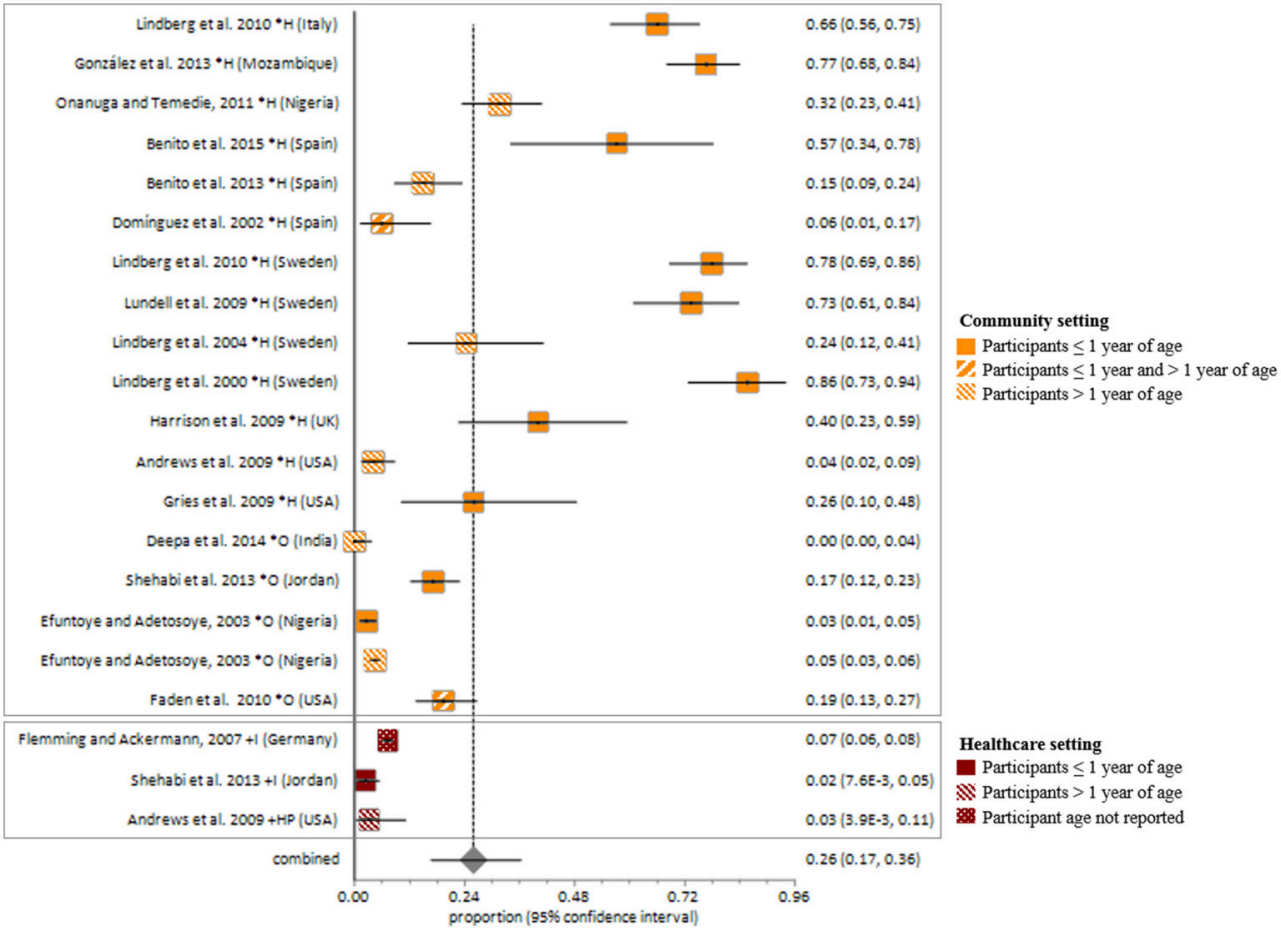
**Meta-analysis of proportions on *S. aureus* fecal carriage rates**. *, Community setting; +, Healthcare setting; H, Healthy participants; O, Outpatients; I, Inpatients; HP, Healthcare personnel.

The pooled random-effects estimate for *S. aureus* fecal carriage within the community and healthcare settings was 26% (95% *CI* = 16.8–36.3; Figure 5). Sub-analyses of *S. aureus* fecal carriage within the community and healthcare settings resulted in pooled random-effects estimates of 31% (95% *CI* = 17.8–46.3) and 5% (95% CI = 1.7–8.9), respectively.

**Figure 6:**
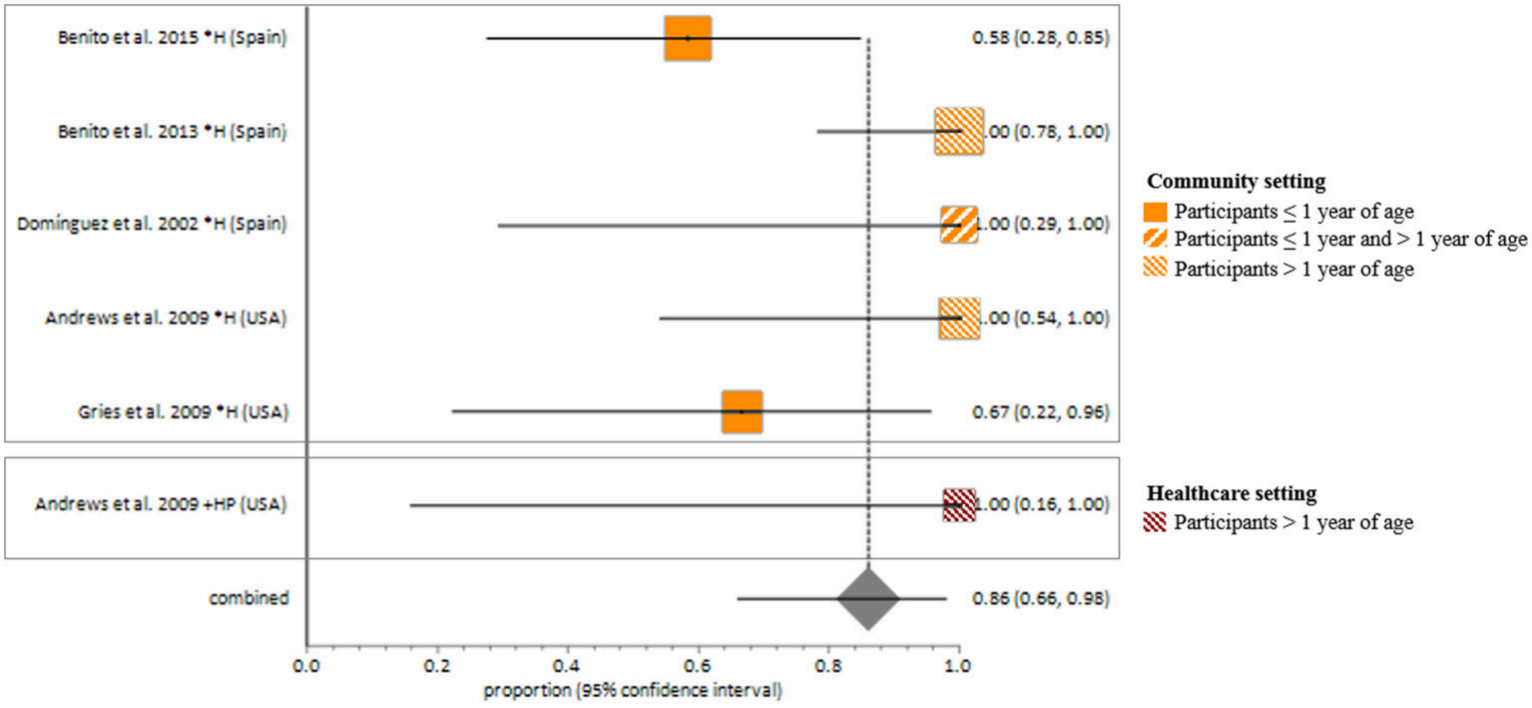
**Meta-analyses of proportions on Methicillin susceptible *Staphylococcus aureus* fecal carriage rates**. Pooled random-effects estimate of MSSA fecal carriage within the community and healthcare setting. *, Community setting; +, Healthcare setting; H, Healthy participants; HP, Healthcare personnel.

**Figure 7:**
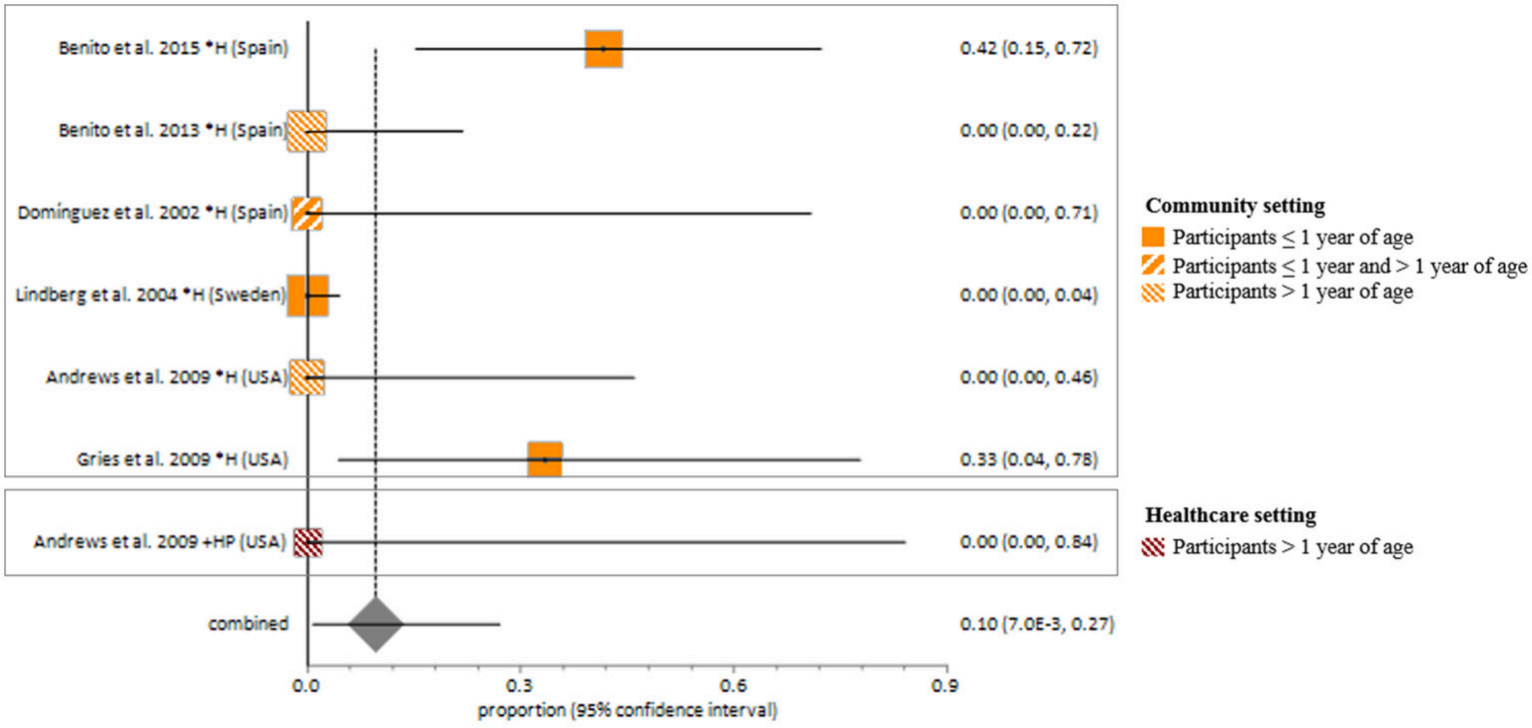
**Meta-analyses of proportions on Methicillin resistant *Staphylococcus aureus* fecal carriage rates**. Pooled random effects estimate of MRSA fecal carriage within community and healthcare settings. *, Community setting; +, Healthcare setting; H, Healthy participants; HP, Healthcare personnel.

MSSA fecal carriage was estimated at 86% (95% *CI* = 65.9–97.9) using the random-effects model (Figure 6). Within the community setting, the random effects estimate for MSSA fecal carriage was 86% (95% *CI* = 62.3–98.5). The pooled random-effects estimates for MRSA fecal carriage were 10% (95% *CI* = 0.7–27.0; Figure 7); and 10% (95% *CI* = 0.4–28.9) within the community setting.

### *S. aureus* fecal carriage rates according to the age of participants

**Figure 8:**
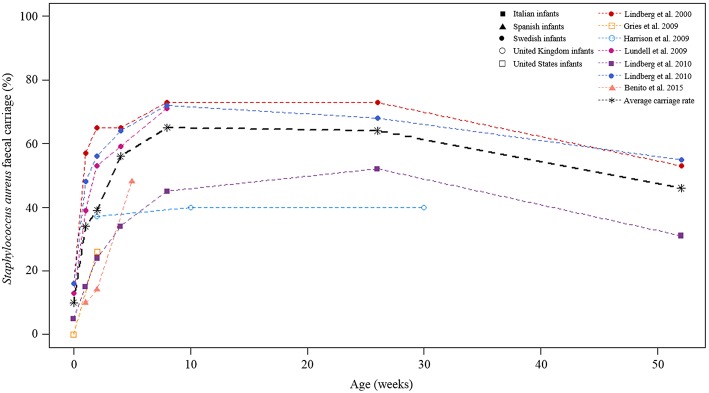
**Longitudinal *S. aureus* fecal carriage rates during the first year of life**. The lines in color indicated fecal carriage rates (%) at each of the time-points measured by the respective studies. The different cohorts are shown by different symbols at each of the time-points studied. The black broken line is the average fecal *S. aureus* carriage rate calculated from all longitudinal studies at the respective time-points under study.

The report on this section is not based on meta-analysis. *S. aureus* fecal carriage rates within the community setting were higher during the first year of life (Figure 8). On average, reports from longitudinal studies revealed an increase in *S. aureus* fecal carriage rates from approximately 10–65% during the first 8 weeks of life (Figure 8). At 6 months of age, the average fecal carriage rate was 64%, thereafter it decreased to approximately 46% at 1 year of life. A longitudinal investigation of fecal MRSA carriage rates from healthy participants from the USA showed an increase in fecal MRSA carriage from 0 to 9% during the first 2 weeks of life (Gries et al., 2009). The highest MRSA fecal carriage rate (23%) reported was from Spanish infants screened at ≤1 year of life (Benito et al., 2015).

### Assessment of antibiotic susceptibility of fecal *S. aureus* isolates

Eight of the 26 eligible studies (31%) included in this review assayed for antibiotic susceptibility of fecal *S. aureus* or MRSA isolates (Table 5). Overall, *S. aureus* or MRSA isolates were screened with 32 different antibiotics across the respective studies using disk diffusion, agar dilution, or the Vitek Legacy System. The use of published guidelines for susceptibility testing were reported by six of the eight studies (Table 5). Susceptibility testing to erythromycin was performed most frequently (88%; 7/8), followed by chloramphenicol, clindamycin, ciprofloxacin, gentamicin, penicillin and vancomycin (75%; 6/8) (Table 5). Vancomycin intermediate or resistant *S. aureus* (VISA/VRSA) were not identified in five of the six studies that screened for vancomycin resistance (Table 5). Only the study by Onanuga and Temedie (2011) reported fecal VRSA carriage of 37% (14/38).

**Table 5:** **Antibiotic resistance profiles across participants screened for fecal *S. aureus* or MRSA**.

**Study**	**Guide-lines applied**	**Techniques applied**	**Total number of *S. aureus* or MRSA isolates screened for resistance**	**Antibiotic resistance profiles of fecal S. aureus or MRSA isolates (%)**																															
				**Aminoglycosides**					**β-lactam/β-lactamase inhibitors**	**Cephalosporin**			**Fluoroquinolones**			**Glycopeptides**		**Lincosamides**	**Lipopeptides**		**Macrolides**		**Oxazolidinones**	**Penicillins**				**Phenicols**	**Pseudomonic acid**	**Pyrimidines**	**Pyrimidines/Sulfonamides**	**Streptogramin**	**Steroidal**	**Tetracyclines**	
				**Amikacin**	**Gentamicin**	**Kanamycin**	**Streptomycin**	**Tobramycin**	**Amoxicillin-clavulanic acid**	**Cefoxitin**	**Cefuroxime**	**Cephalothin**	**Ciprofloxacin**	**Levofloxacin**	**Ofloxacin**	**Teicoplanin**	**Vancomycin**	**Clindamycin**	**Colistin**	**Polymyxin B**	**Erythromycin**	**Spiramycin**	**Linezolid**	**Ampicillin**	**Methicillin**	**Penicillin**	**Oxacillin**	**Chloramphenicol**	**Mupirocin**	**Trimethoprim**	**Co-trimoxazole**	**Virginiamycin**	**Fusidic acid**	**Doxycycline**	**Tetracycline**
Domínguez et al., 2002	NR	Agar dilution method	3		0	0	0	0					0			0	0	0			67					100	0	0							0
Efuntoye and Adetosoye, 2003	NCCLS	Disk diffusion method	72			22						0						9	99	99	67			79	7	100		0							
Lindberg et al., 2004b	SRGA	Disk diffusion method	116		0			0					0			0	0	1			3					78	0	0		0			1		2
Flemming and Ackermann, 2007	NR	Disk diffusion method	198																								15								
Srinivasan et al., 2010	CLSI	Vitek Legacy System	31^*^		3					100			0	32			0	32			81					100			0		3				
Onanuga and Temedie, 2011	CLSI	Disk diffusion and agar dilution method	38	0	5				18	34	24		8		8		37				34	0		68				34			37	0		61	
Benito et al., 2013	CLSI CA-SFM	Disk diffusion method	15		0	0	0	7		0			0				0	20			20		0			60	0	0	7		0		0		0
Benito et al., 2015	CLSI EUCAST	Disk diffusion method	25		36	0	16	36		40			4				0	32			36		0			40	40	0	0		0		4		0

(CA-SFM), Antibiogram Committee of the French Society of Microbiology; (CLSI), Clinical Laboratory Standards Institute; (EUCAST), European Committee on Antimicrobial Susceptibility; (NCCLS), National Committee on Clinical Laboratory Standards; (SRGA), Swedish Reference Group for Antibiotics; NR, Not reported.

* Number of MRSA isolates (detected using oxacillin screening agar) screened for antibiotic resistance

Antibiotic resistance rates (%): 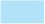 0; 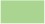 > 0–10; 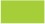 > 10–20; 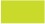 > 20–30; 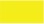 > 30–40; 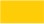 > 40–50; 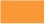 > 50–60; 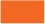 > 60–70; 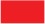 > 70–80; 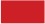 > 80–90; 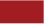 > 90–100.

### Genotyping of *S. aureus* isolated from fecal specimens

Techniques used to genotype *S. aureus* isolated from fecal specimens included multiple-locus variable-number tandem repeat analysis (MLVA), pulsed-field gel electrophoresis (PFGE), random amplified polymorphic DNA (RAPD) analysis, staphylococcal cassette chromosome *mec* (SCC*mec*), accessory gene regulator (*agr*) and *Staphylococcus aureus* protein A (*spa*) typing (Tables 3, 4). Genotyping was performed in slightly more reports from the healthcare setting (67%; 8/12) compared to the community (58%; 11/19). Gel-based methods (PFGE, RAPD and MLVA) were employed in 58% (7/12) and 42% (8/19) of studies in the healthcare and community settings, respectively. In addition, similar rates (26% vs. 25%) in the use of sequence-based methods (*spa* typing, SCC*mec* typing and MLST) for genotyping of *S. aureus* strains were reported from community and healthcare settings. Only a single study conducted in a developing country (Jordan) performed genotyping of the *S. aureus* strains (Shehabi et al., 2013).

### Assessment of the detection of *S. aureus* virulence genes

Virulence genes screened included the aureolysin enzyme, biofilm-associated protein, collagen-binding protein, exfoliative toxins (ETs), staphylococcal enterotoxins (SEs), toxic shock syndrome toxin (TSST), and Panton-Valentine leukocidin (PVL) (Tables 3, 4). More community–based investigations screened for *S. aureus* virulence genes compared to reports from the healthcare setting. Thus, 53% (10/19), 37% (7/19), and 37% (7/19) of studies conducted in the community setting reported on TSSTs, SEs, ETs, respectively, using PCR, reverse passive latex agglutination tests or enzyme-linked immunosorbent assays. Approximately one third of the studies conducted in the community setting (6/19) reported on PCR detection of the PVL genes. In studies conducted in the healthcare setting; 8% (1/12), 25% (3/12.), 8% (1/12), and 25% (3/12) reported on TSSTs, SEs, ETs, and PVL, respectively.

### *S. aureus* and MRSA fecal carriage as risk factors for disease development

Two studies included in this review identified enterotoxin producing *S. aureus* strains from fecal specimens of patients with diarrhea (Efuntoye and Adetosoye, 2003; Flemming and Ackermann, 2007). Another study reported that all patients colonized with MRSA in both the nares and rectum (8/8) developed an infection (Srinivasan et al., 2010). In addition, two of the nine patients, colonized with MRSA in the rectum only, were concurrently or subsequently infected. *Spa* typing on a subset of colonizing isolates from the nares and rectum noted that the majority (69%; 9/13) were clonally related to infecting isolates (Srinivasan et al., 2010). In support of the potential of fecal carriage for infection, it has also been shown that *S. aureus* detection occurs more frequently from rectal specimens of children with skin and soft tissue abscesses (47%; 28/60) compared with the control group (1%; 1/90) (*P* = 0.0001) (Faden et al., 2010).

## Discussion

Our results clearly showed that fecal *S. aureus* carriage from healthy infants is high during the first year of life. Specifically, *S. aureus* fecal carriage rates increased during the first 8 weeks of life followed by a gradual decrease towards 1 year of life. The reasons for this abrupt increase in fecal carriage very early in life (especially from healthy infants) is not yet clear, however a potential explanation may be early life care-giving practices, particularly breastfeeding. For example, colostrum contains the highest levels of human milk oligosaccharides (HMOs) (Bode, 2012), which have been suggested to stimulate *S. aureus* growth (Hunt et al., 2012). Moreover, *S. aureus* strains may be transmitted from parents via skin contact (Lindberg et al., 2004a) or from the mother via breastfeeding (Kawada et al., 2003; Lindberg et al., 2004a; Benito et al., 2015). Furthermore, staphylococci from the maternal GIT or skin surrounding the areola may be transferred to breast milk during lactation (Thum et al., 2012; Fernández et al., 2013). Higher *S. aureus* fecal carriage rates have also been noted from breast-fed in comparison to formula-fed or mixed-fed infants (González et al., 2013; Salminen et al., 2015). The observed change in the dynamics of *S. aureus* fecal carriage after 8 weeks of life may be explained by the increase in anaerobic bacteria from around 1 week of life (Bezirtzoglou, 1997; Adlerberth et al., 2006; Adlerberth and Wold, 2009; Jost et al., 2012), as well as the introduction of formula feeding (González et al., 2013) and solid foods (Bergström et al., 2014; Voreades et al., 2014). Infant fecal bacterial profiles have also been shown to change during the course of the lactation period (Cabrera-Rubio et al., 2012; González et al., 2013).

This systematic review does not only provide insight into the dynamics of fecal *S. aureus* carriage rates during the first year of life; but also highlights that *S. aureus* and MRSA fecal carriage is a potential risk factor for subsequent infections. Vancomycin is regarded as one of the drugs of choice for MRSA infections (Tarai et al., 2013); however the emergence of vancomycin resistant *S. aureus* (VRSA) poses yet another threat to infection control (Hiramatsu, 1998; Spagnolo et al., 2014). The intestinal tract, in particular, may be a key potential reservoir for the emergence and transmission of VRSA isolates due to the intestinal coexistence (Ray et al., 2003), and potential transfer of the *vanA* gene from VRE to MRSA (Courvalin, 2006). Although, 23% of the studies included in this review screened for fecal carriage of VRSA within community and healthcare settings (Domínguez et al., 2002; Lindberg et al., 2004b; Srinivasan et al., 2010; Onanuga and Temedie, 2011; Benito et al., 2013, 2015); only a single study, performed in Nigeria, reported VRSA fecal carriage (Onanuga and Temedie, 2011). It is noteworthy, however, that this finding should be interpreted with caution as the disk diffusion method was used to screen for vancomycin resistance at 30 μg/ml, which is not recommended by the CLSI guidelines (Clinical Laboratory Standards Institute, 2012).

Healthcare associated fecal screening for *S. aureus* and MRSA is of key importance in infection control (Campillo et al., 2001; Ray et al., 2003; Bhalla et al., 2007). For example, it has been shown that select staphylococcal enterotoxins (SEs) may contribute to the colonizing success of *S. aureus* strains in the GIT (Nowrouzian et al., 2011), which could potentially facilitate in its transmission. Moreover, *S. aureus* and MRSA fecal carriage may complicate de-colonization, with a potential to contribute to infections within the healthcare setting (Campillo et al., 2001; Dupeyron et al., 2002; Ray et al., 2003; Srinivasan et al., 2010). To prevent nosocomial transmission and infection, two recent studies (Roth et al., 2016; Senn et al., 2016) have also highlighted the importance of screening for *S. aureus* fecal carriage on admission in the following risk groups: patients admitted to surgery or intensive care units with a history of MRSA colonization or infection; hospitalization during the past year; or direct transfer from another healthcare facility. Only a single study was considered eligible for inclusion in our meta-analyses of the proportions on MSSA and MRSA fecal carriage within the healthcare setting. Therefore we could not determine the fecal carriage rate for MSSA or MRSA within this setting.

A major limitation in this systematic review is the poor study design and limited data available from studies assessing the fecal carriage rates of *S. aureus* and MRSA. For example, a large proportion of potentially eligible articles were excluded due to the lack of information regarding participants' contact with healthcare facilities as well as the duration of hospital admission prior to *S. aureus* and MRSA screening. This information is essential in comparing fecal carriage rates from community and healthcare settings. Furthermore, a number of studies could not be included in calculating the pooled estimates for MSSA and MRSA fecal carriage (from both community and healthcare settings) due to the lack of molecular techniques incorporated to confirm MRSA carriage. On the other hand, the extent in which our observations could have changed if unavailable articles were included is unclear. However, based on the rigorous appraisal of various studies in this systematic review, we conclude that the excluded articles are not likely to impact significantly on observations presented in the manuscript. In addition, more studies from both developed and developing countries are needed in order to determine *S. aureus* and MRSA fecal carriage and transmission within and between the community and healthcare settings. In support of this, rural areas and low socioeconomic status have been shown to contribute to higher fecal transmission rates of *S. aureus* and MRSA (Vale and Vítor, 2010). Finally, there is the need for more sequence-based genotyping data on *S. aureus* and MRSA fecal carriage as the majority of studies from developed countries made use of gel-based methods which are not ideal when comparing isolates on a global level.

## Conclusion

*S. aureus*, MSSA and MRSA fecal carriage rates within both the community and healthcare setting are not negligible and estimated at 26, 86, and 10%, respectively. Therefore, preventative strategies which include fecal *S. aureus* screening of high risk patients are necessary for infection control within these settings. More studies are needed to determine the role of fecal *S. aureus* carriage as a risk factor for disease development; as well as fecal carriage rates of MSSA, MRSA, and VRSA from both community and healthcare settings. Furthermore, well-structured research should be conducted and sequence-based genotyping techniques should be employed. The latter will allow for comparison of isolates on a global level in both developing and developed countries.

## Author contributions

MK and SC initiated the project. SC, MRN, and MK searched the databases for potentially eligible articles based on their titles and abstracts. SC extracted the data and contacted authors of potentially eligible publications to obtain healthcare information on participants when this information was unclear or not provided by the articles. SC, MK, and AS reviewed the articles. SC, LT, and MK performed the statistical analysis and interpreted the results. SC, LT, AS, MPN, and MK wrote the manuscript. All the authors reviewed the final version of the manuscript prior to submission for publication.

## Funding

This work was supported by the Bill and Melinda Gates Foundation Global Health Grant (OPP1017641), the National Research Foundation (South Africa), the Carnegie Corporation of New York (United States of America), the US National Institutes of Health (1U01AI110466-01A1), and the Wellcome Trust, United Kingdom (102429/Z/13/Z).

## Role of funding source

Any opinions, findings and conclusions, or recommendations expressed in this review are those of the authors, and therefore do not represent the official position of the funders. The funders had no role in the study design, data collection and analysis, decision to publish, or preparation of the manuscript. The first and the corresponding author had full access to the study data. All authors had final responsibility for the decision to submit the article for publication.

### Conflict of interest statement

The authors declare that the research was conducted in the absence of any commercial or financial relationships that could be construed as a potential conflict of interest.
